# Fat-to-muscle ratio is a useful index for cardiometabolic risks: A population-based observational study

**DOI:** 10.1371/journal.pone.0214994

**Published:** 2019-04-09

**Authors:** Yuan-Yuei Chen, Wen-Hui Fang, Chung-Ching Wang, Tung-Wei Kao, Hui-Fang Yang, Chen-Jung Wu, Yu-Shan Sun, Ying-Chuan Wang, Wei-Liang Chen

**Affiliations:** 1 Department of Internal Medicine, Tri-Service General Hospital Songshan Branch, Taipei, Taiwan, Republic of China; 2 Division of Family Medicine, Department of Family and Community Medicine, Tri-Service General Hospital, School of Medicine, National Defense Medical Center, Taipei, Taiwan, Republic of China; 3 Division of Geriatric Medicine, Department of Family and Community Medicine, Tri-Service General Hospital, School of Medicine, National Defense Medical Center, Taipei, Taiwan, Republic of China; 4 Graduate Institute of Clinical Medical, College of Medicine, National Taiwan University, Taipei, Taiwan, Republic of China; 5 Division of Family Medicine, Department of Community Medicine, Taoyuan Armed Forces General Hospital, Taoyuan, Taiwan, Republic of China; Beijing Key Laboratory of Diabetes Prevention and Research, CHINA

## Abstract

Metabolic disorders are prevalent worldwide and have recently become public health problems recently. Previous studies have proposed different body composition indices for predicting future cardiovascular risks. We hypothesized an association among fat-to-muscle ratio (FMR), metabolic syndrome (MetS), hypertension (HTN), prediabetes, type 2 diabetes mellitus (DM), and cardiovascular risk in an adult population. A total of 66829 eligible subjects composed of 34182 males and 32647 females aged 20 years or older were obtained from health examinations in the Tri-Service General Hospital from 2011 to 2017. The body composition indices included fat and muscle mass measured by bioelectrical impedance analysis. A multivariable regression model was performed in a large population-based cross-sectional study. FMR was significantly associated with MetS, prediabetes, DM and HTN in all models of both genders. Based on quartile analysis, higher FMR had higher predictive ability for adverse health outcomes. The association between different definitions of MetS and the Framingham risk score was analyzed, and FMR-incorporated MetS was more useful for predicting higher Framingham risk scores than traditional definitions. FMR was a useful indicator for the presence of adverse cardiometabolic risks. Compared to traditional definition of MetS, FMR-incorporated MetS had a greater ability to predict incident cardiovascular risks. FMR seemed to be a simple and effective index for the early prevention and management of cardiometabolic events.

## Introduction

The current worldwide prevalence of obesity has increased progressively. As a major public health problem in the world, an increasing number of individuals have been diagnosed with obesity and metabolic syndrome (MetS) in Taiwan with high risks for the development of diabetes mellitus (DM) and hypertension (HTN)[1]. An emerging concept called “sarcopenic obesity”, which reflect a combination of age-associated skeletal muscle loss and fat mass accumulation[2], was also recognized as a critical public health risk in the aging society. Previous studies have proposed an association between sarcopenic obesity and MetS in both sexes[3] and between sarcopenic obesity and insulin resistance in the adult population[4].

Increased total fat mass and its distribution were significantly associated with insulin resistance, glucose intolerance and high risks of DM and cardiovascular diseases[5], wthile loss of skeletal muscle was reported to contribute to MetS and DM in the adult population[6, 7]. However, the associations among simultaneous skeletal muscle mass loss, fat mass accumulation and metabolic disorders have not been well established. The ratio of visceral fat to thigh muscle area was considered as a single anthropometric index for insulin resistance and glucose metabolism[8]. Park et al. suggested muscle-to-fat ratio as a useful indicator for predicting MetS[9].

Although different types of body composition indices have valid predictions for metabolic dysfunction, there is no comprehensive index that can be used simultaneously for the risk of cardiometabolic disorders. The objective of this cross-cohort analysis was to critically examine whether fat-to-muscle ratio (FMR) was associated with the presence of MetS, prediabetes, DM and HTN and to develop sound definitions of MetS.

## Methods

### Study design and participants

**Fig 1:**
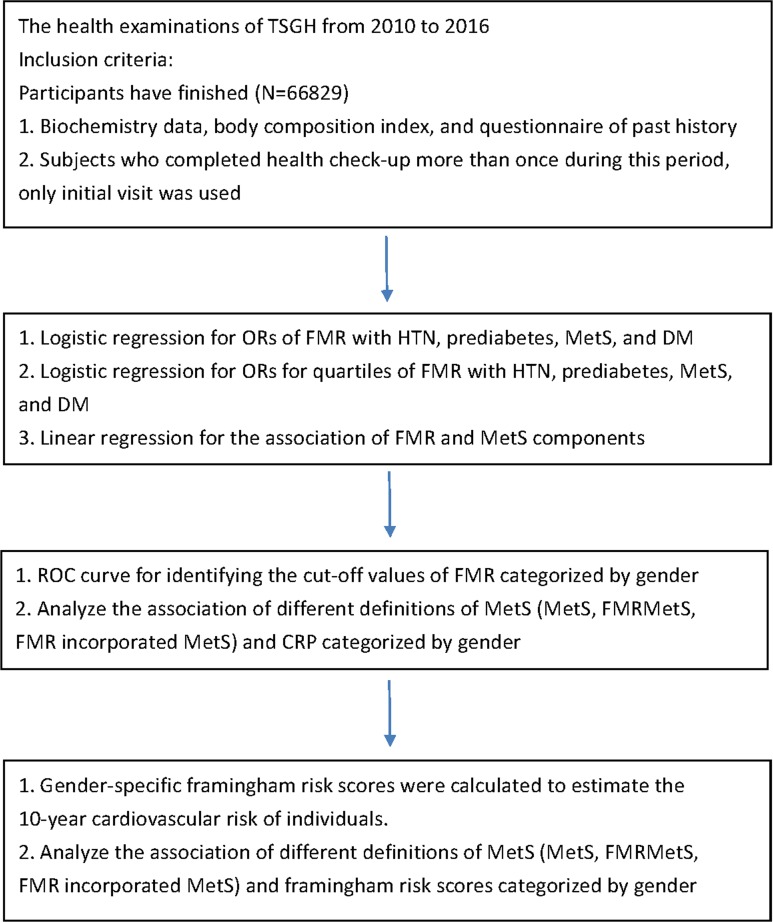
Flow chart which represented the steps of analysis performed in the study.

All data were derived from health examinations in the Tri-Service General Hospital from 2010 to 2016. The study design met the requirements of the Helsinki Declaration and the design was approved by the institutional review board of Tri-Service General Hospital. Because the data were analyzed anonymously, the institutional review board of Tri-Service General Hospital waived the need to acquire individual informed consent. Based on the flow chart of the study shown in Fig 1, subjects who attended the health check-up and finished comprehensive examinations, including laboratory biochemistry tests, body composition exams and questionnaires of the personal history were included in this study. 66829 eligible subjects were analyzed in a step-by-step manner in the following orders. First, the ORs of FMR in males and females for the presence of MetS, prediabetes, DM and HTN were conducted by multivariate logistic regression. Next, FMR was divided into quartiles to analyze its association with the presence of adverse health outcomes. Third, multivariable linear regression was used to assess the association between FMR and individual MetS components. Last, we calculated the optimal cut-off values of FMR for MetS in both genders and then created different definitions of MetS to compare the effect of inflammatory process with the traditional MetS criteria. In addition, we analyzed the association between different definitions of MetS and the Framingham risk score by using multivariable linear regression.

### Measurement of body composition

Percentage of skeletal muscle mass and percentage of body fat were measured by bioelectric impedance analysis (BIA) (InBody720, Biospace, Inc., Cerritos, CA, USA) in the present study. BIA has been proven to be one of the most practical procedures to estimate body composition among different groups because of its ready accessibility, quick assessment, low cost, and its high validity against DEXA as the reference method[10]. FMR was defined as the ratio of fat mass to lean muscle mass.

### General definition of MetS

According to the Taiwan Health Promotion Administration of the Ministry of Health and Welfare in 2007, the diagnosis of MetS was defined if an individual manifested 3 or more of the following components: (1) waist circumference>90 cm for male participants and >80 cm for female participants.; (2) systolic blood pressure≥130 mmHg, diastolic blood pressure≥80 mmHg, or self-reported hypertension (3) triglyceride≥150 mg/dL (1.7 mmol/L); (4) fasting plasma glucose≥100 mg/dL, a past history of diabetes status, or the use of antidiabetic agents; and (5) HDL-C<40 mg/dL (1.03 mmol/L) for male participants and <50 mg/dL (1.3 mmol/L) for female participants.

### Different definitions of MetS

In our study, we created two different definitions of MetS to compare the effects of the inflammatory process with the traditional MetS. To assess the cut-off values of FMR for MetS, a receiver operating characteristic (ROC) curve analysis was performed. In males, the AUROC value was 0.673 (95%CI: 0.660–0.686), and the optimal cut-off value was 0.76 using the maximal Youden index, with a sensitivity of 0.763 and a specificity of 0.494. In females, the AUROC value was 0.701 (95%CI: 0.685–0.717), and the optimal cut-off value was 1.51 with a sensitivity of 0.792 and a specificity of 0.509. Subjects who had FMR above the cut-off values (males: 0.76; females: 1.51) were categorized as “MetFMR”.

First, “FMRMetS” was defined as participants with “MetFMR” along with at least two of four components of MetS except waist circumference. Second, “FMR incorporated MetS” was defined as MetFMR along with at least three out of five components of MetS.

### Definition of Type 2 DM

Type 2 DM was defined base on the American Diabetes Association criteria as follows: fasting plasma glucose ≥126 mg/dL; hemoglobin A1c test ≥6.5%; random plasma glucose ≥200 mg/dL; and past history of diabetes status, or use of antidiabetic agents[11].

### Definition of HTN

Based on the guidelines of the Taiwan Society of Cardiology and the Taiwan Hypertension Society for the management of hypertension, HTN was defined as blood pressure being higher than 140/90 mmHg or subjects taking antihypertensive agents[12].

### Measurement o covariates

The regular health examinations included standard evaluations of comprehensive biochemistry tests and anthropometric measurements. The body mass index (BMI) was obtained based on the formula in which the weight of the subject in kilograms is divided by the square of their height in meters(kg/m^2^). The waist circumference was measured at the mid-level between the iliac crest and the lower border of the 12^th^ rib while the subject stood with feet 25–30 cm apart. Hemodynamic status included systolic blood pressure (SBP) and diastolic blood pressure (DBP) estimated when the participants were seated. Biochemical analysis was conducted by drawing blood samples from subjects after fasting for at least 8 hours. The fasting plasma glucose (FPG) was detected using a glucose oxidase method. Serum levels of lipid profiles such as total cholesterol (TC), triglycerides (TG) and high-density lipoprotein cholesterol (HDL-C), were measured using an enzymatic colorimetric method.

### Statistical analysis

All statistical estimations were performed using the Statistical Package for the Social Sciences, version18.0 (SPSS Inc., Chicago, IL, USA) for Windows. Student’s t-tests and Pearson's chi-square tests were performed to examine the differences between the gender groups in terms of demographic information and laboratory data. A two-sided *p*-value of ≤ 0.05 was regarded as the threshold for statistical significance. The extend-model approach was performed in the study with multivariable adjustment for pertinent clinical variables. Linear regression with beta coefficients was conducted for the association of FMR with MetS components, inflammation and the Framingham risk score. Logistic regression for ORs was used to examine the association between FMR and the presence of MetS, prediabetes, DM and HTN in a cross-sectional analysis. A receiver operating characteristic (ROC) curve analysis was calculated for the area under the ROC (AUROC), 95% confidence intervals (CI), sensitivity and specificity to assess the cut-off values of FMR.

## Results

### Characteristics of the study population

All data were obtained from the annual health examinations conducted in the Tri-Service General Hospital (TSGH) from 2010 to 2016. There were 34182 eligible males and 32647 eligible females enrolled in the study after excluding those with missing data. The mean age of male subjects was 42.35±16.14 years old, and the mean age of female was 42.63±15.95 years old. The prevalence of MetS, FMRMetS, and FMR-incorporated MetS were significantly higher in males than females (P<0.05). All demographic characteristics listed in Table 1, such as body composition index, components of MetS and laboratory biochemistry data, had significant difference.

**Table 1:** Characteristics of entire groups of participants with or without metabolic syndrome.

Variables	Male	Female	P-value
**Continuous Variables, mean (SD)**			
** Age**	42.35 (16.14)	42.63 (15.95)	0.029
** Percentage of lean mass (%)**	31.03 (4.60)	20.74 (3.05)	<0.001
** PBF (%)**	25 (6.33)	31.94 (6.66)	<0.001
** FMR**	0.82 (0.24)	1.57 (0.38)	<0.001
** BMI (kg/m**^**2**^**)**	24.93 (3.80)	22.94 (4.09)	<0.001
** WC (cm)**	84.63 (9.90)	74.46 (10.36)	<0.001
** SBP (mmHg)**	123.58 (16.11)	115.09 (17.84)	<0.001
** DBP (mmHg)**	77.18 (11.76)	71.40 (11.41)	<0.001
** TG (mg/dL)**	129.82 (106.55)	95.35 (63.51)	<0.001
** HDL-C (mg/dL)**	48.67 (11.67)	60.55 (14.11)	<0.001
** FPG (mg/dL)**	95.09 (23.28)	91.67 (19.91)	<0.001
** TC (mg/dL)**	183.38 (35.14)	185.66 (35.50)	<0.001
** UA (mg/dL)**	6.39 (1.32)	4.74 (1.09)	<0.001
** Cr (mg/dL)**	0.96 (0.35)	0.68 (0.21)	<0.001
** AST (mg/dL)**	22.47 (14.55)	18.99 (13.58)	<0.001
** Albumin (mg/dL)**	4.54 (0.31)	4.43 (0.29)	<0.001
** hsCRP (mg/dL)**	0.25 (0.54)	0.21 (0.42)	<0.001
**Category Variables, (%)**			
** Framingham Score**	6.97 (7.63)	1.18 (2.65)	<0.001
** MetS**	4106 (65.5)	16696 (50.8)	<0.001
** FMRMetS**	1981 (66.9)	5504 (51.5)	<0.001
** FMR-incorporated MetS**	1500 (64.7)	5975 (52.8)	<0.001
** Proteinuria**	8597 (29.9)	7213 (29.0)	0.004
** Smoking**	4971 (42.0)	712 (9.0)	0.006
** Drinking**	6103 (60.9)	1868 (28.4)	0.007

PBF, percentage body fat; FMR, fat-muscle ratio; BMI, body mass index; WC, waist circumference; SBP, systolic blood pressure; DBP, diastolic blood pressure; TG, triglyceride; HDL-C, high density lipoprotein cholesterol; FPG, fasting plasma glucose; TC, total cholesterol; UA, uric acid; Cr, creatinine; AST, aspartate aminotransferase; hsCRP, high sensitivity C-reactive protein

### Association between FMR and the presence of MetS, Prediabetes, DM and HTN

Table 2 represents the odd ratios (ORs) of the presence of MetS, prediabetes, DM and HTN in male and female participants with FMR. The ORs of MetS were higher than other adverse health outcomes in all adjusted models of male (ORs = 5.69, 4.63, 4.20; 95%CI = 4.05–7.99, 3.26–6.56, 2.91–6.05, respectively). In females, the ORs of MetS and DM were similar in all models. The FMR tended to have more predictive ability for the presence of DM in fully adjusted model (ORs = 1.92; 95%CI = 1.14–3.23).

**Table 2:** Association between fat-muscle ratio and the presence of MetS, prediabetes, DM and HTN.

Sex	Variable	Model ^a^ 1 OR (95% CI)	*P* Value	Model ^a^ 2 OR (95% CI)	*P* Value	Model ^a^ 3 OR (95% CI)	*P* Value
**Male**	**MetS**	5.69 (4.05–7.99)	<0.001	4.63 (3.26–6.56)	<0.001	4.20 (2.91–6.05)	<0.001
	**Prediabetes**	1.85 (1.28–2.68)	<0.001	1.50 (1.02–2.19)	0.037	1.53 (1.04–2.23)	0.030
	**DM**	2.35 (1.39–3.99)	0.002	2.30 (1.35–3.93)	0.002	2.37 (1.38–4.06)	0.002
	**HTN**	3.21 (2.28–4.53)	<0.001	2.68 (1.89–3.80)	<0.001	2.70 (1.91–3.83)	<0.001
**Female**	**MetS**	2.59 (1.96–3.42)	<0.001	1.92 (1.42–2.59)	<0.001	1.86 (1.36–2.56)	<0.001
	**Prediabetes**	2.00 (1.47–2.72)	<0.001	1.62 (1.17–2.23)	0.003	1.63 (1.18–2.25)	0.003
	**DM**	2.31 (1.40–3.81)	<0.001	1.92 (1.14–3.24)	0.014	1.92 (1.14–3.23)	0.015
	**HTN**	1.67 (1.24–2.26)	<0.001	1.44 (1.05–1.97)	0.023	1.45 (1.06–1.98)	0.021

^a^ Adjusted covariates:

Model 1 = age

Model 2 = Model 1 + proteinuria,TC, UA, Cr, AST, albumin, hsCRP

Model 3 = Model 2 + history of smoking, drinking

### Association between quartiles of FMR and the presence of MetS, Prediabetes, DM and HTN

In Table 3, the FMR in each gender was divided into quartiles and the higher quartiles (Q2, Q3 and Q4) were compared to baseline (Q1) in subgroups to analyze the association between the FMR and the presence of adverse health outcomes. The intervals of FMR in quartiles were <0.66, 0.66–0.81, 0.81–0.96, and >0.96 in males and <1.30, 1.30–1.55, 1.55–1.80, and >1.80 in females from Q1 to Q4, respectively. Obviously, the higher quartile of FMR had more predictive ability for the presence of MetS, prediabetes, DM and HTN in male and female participants.

**Table 3:** Association between fat-muscle ratio in quartiles and the presence of MetS, prediabetes, DM and HTN.

Sex	Variable	Quartiles	Model ^a^ 1 OR (95% CI)	*P* Value	Model ^a^ 2 OR (95% CI)	*P* Value	Model ^a^ 3 OR (95% CI)	*P* Value
**Male**	**MetS**	Q2 v.s. Q1	2.47 (1.88–3.25)	<0.001	2.17 (1.65–2.87)	<0.001	2.19 (1.66–2.90)	<0.001
		Q3 v.s. Q1	3.11 (2.39–4.07)	<0.001	2.54 (1.93–3.34)	<0.001	2.57 (1.95–3.38)	<0.001
		Q4 v.s. Q1	4.48 (3.45–5.81)	<0.001	3.45 (2.63–4.53)	<0.001	3.54 (2.69–4.66)	<0.001
	**Prediabetes**	Q2 v.s. Q1	1.67 (1.25–2.23)	<0.001	1.40 (1.04–1.88)	0.028	1.40 (1.04–1.89)	0.026
		Q3 v.s. Q1	1.98 (1.49–2.62)	<0.001	1.39 (1.03–1.86)	0.030	1.39 (1.04–1.87)	0.028
		Q4 v.s. Q1	2.50 (1.90–3.28)	<0.001	1.50 (1.12–2.01)	0.006	1.51 (1.13–2.03)	0.005
	**DM**	Q2 v.s. Q1	1.40 (0.85–2.30)	0.183	1.27 (0.77–2.11)	0.355	1.26 (0.76–2.09)	0.377
		Q3 v.s. Q1	2.20 (1.39–3.49)	<0.001	1.75 (1.09–2.82)	0.022	1.73 (1.08–2.80)	0.024
		Q4 v.s. Q1	3.21 (2.07–5.00)	<0.001	1.99 (1.25–3.18)	0.004	1.99 (1.25–3.19)	0.004
	**HTN**	Q2 v.s. Q1	1.42 (1.09–1.86)	0.010	1.24 (0.95–1.63)	0.115	1.26 (0.96–1.65)	0.101
		Q3 v.s. Q1	2.22 (1.72–2.87)	<0.001	1.74 (1.34–2.26)	<0.001	1.76 (1.35–2.28)	<0.001
		Q4 v.s. Q1	2.82 (2.20–3.61)	<0.001	2.00 (1.55–2.60)	<0.001	2.03 (1.56–2.64)	<0.001
**Female**	**MetS**	Q2 v.s. Q1	2.27 (1.50–3.45)	<0.001	1.63 (1.05–2.52)	0.028	1.65 (1.06–2.54)	0.025
		Q3 v.s. Q1	3.94 (2.66–5.84)	<0.001	2.30 (1.52–3.50)	<0.001	2.31 (1.52–3.51)	<0.001
		Q4 v.s. Q1	6.12 (4.17–8.99)	<0.001	2.48 (1.63–3.77)	<0.001	2.51 (1.65–3.82)	<0.001
	**Prediabetes**	Q2 v.s. Q1	1.80 (1.10–2.95)	0.020	1.35 (0.82–2.23)	0.243	1.36 (0.82–2.26)	0.228
		Q3 v.s. Q1	3.77 (2.39–5.93)	<0.001	2.35 (1.47–3.75)	<0.001	2.36 (1.47–3.77)	<0.001
		Q4 v.s. Q1	5.36 (3.44–8.34)	<0.001	2.40 (1.50–3.84)	<0.001	2.42 (1.51–3.88)	<0.001
	**DM**	Q2 v.s. Q1	4.62 (1.36–15.71)	0.014	3.61 (1.04–12.54)	0.043	3.64 (1.05–12.64)	0.042
		Q3 v.s. Q1	7.38 (2.25–24.19)	<0.001	4.56 (1.35–15.33)	0.014	4.60 (1.37–15.48)	0.014
		Q4 v.s. Q1	12.18 (3.80–39.05)	<0.001	4.76 (1.43–15.88)	0.011	4.79 (1.43–15.99)	0.011
	**HTN**	Q2 v.s. Q1	1.76 (1.14–2.71)	0.010	1.30 (0.83–2.04)	0.244	1.31 (0.84–2.04)	0.243
		Q3 v.s. Q1	2.64 (1.75–3.97)	<0.001	1.60 (1.04–2.45)	0.033	1.58 (1.03–2.43)	0.035
		Q4 v.s. Q1	4.61 (3.11–6.83)	<0.001	1.94 (1.27–2.97)	0.002	1.95 (1.28–2.99)	0.002

^a^ Adjusted covariates:

Model 1 = age

Model 2 = Model 1 + proteinuria, TC, UA, Cr, AST, albumin, hsCRP

Model 3 = Model 2 + history of smoking, drinking

### Association between different definitions of MetS and Framingham risk score

We analyzed the association of MetS, FMRMetS and FMR-incorporated MetS with the Framingham risk score listed in Table 4. All definitions of MetS had significant association with increased Framingham risk score. FMR-incorporated MetS (β = 3.64, 95%CI = 3.25–4.03) was more closely associated with the Framingham risk score than MetS (β = 3.59, 95%CI = 3.26–3.92) in the fully adjusted model in males. However, in females, not only FMR-incorporated MetS (fully adjusted model: β = 2.10, 95%CI = 1.84–2.35) but also FMRMetS (fully adjusted model: β = 1.90, 95%CI = 1.66–2.15) were more closely associated with the Framingham risk score than MetS (fully adjusted model: β = 1.74, 95%CI = 1.53–1.96) in all models.

**Table 4:** Association between the Framingham risk score and different definitions of MetS.

		Model ^a^ 1 β^b^ (95% CI)	*P* Value	Model ^a^ 2 β^b^ (95% CI)	*P* Value	Model ^a^ 3 β^b^ (95% CI)	*P* Value
Male	**MetS**	4.47 (4.06–4.89)	<0.001	4.24 (3.84–4.63)	<0.001	3.59 (3.26–3.92)	<0.001
	**FMRMetS**	4.01 (3.54–4.47)	<0.001	3.73 (3.29–4.17)	<0.001	3.25 (2.89–3.61)	<0.001
	**FMR + MetS**	4.29 (3.78–4.79)	<0.001	4.15 (3.67–4.63)	<0.001	3.64 (3.25–4.03)	<0.001
Female	**MetS**	1.86 (1.64–2.07)	<0.001	1.76 (1.54–1.98)	<0.001	1.74 (1.53–1.96)	<0.001
	**FMRMetS**	2.05 (1.80–2.29)	<0.001	1.95 (1.70–2.20)	<0.001	1.90 (1.66–2.15)	<0.001
	**FMR + MetS**	2.23 (1.97–2.49)	<0.001	2.15 (1.88–2.41)	<0.001	2.10 (1.84–2.35)	<0.001

^a^ Adjusted covariates:

Model 1 = age

Model 2 = Model 1 + proteinuria, TC, UA, Cr, AST, albumin, hsCRP

Model 3 = Model 2 + history of smoking, drinking

### Association between FMR and individual components of MetS

Multivariable linear regressions of FMR and MetS components performed with the adjusted extend-model approach are shown in S1 Table. As expected, the FMR was significantly associated with higher blood pressure, central obesity, hypertriglyceridemia, hyperglycemia and lower HDL.

### Association between different definitions of MetS with inflammation

Multivariable beta coefficients regression was performed for the association between different definitions of MetS and levels of CRP, as shown in S2 Table. It was surprising that different definitions of MetS including MetS, MetFMR, FMRMetS and FMR-incorporated MetS had significant associations with increased levels of CRP in both sexes, except MetS in the fully adjusted model in males.

## Discussion

In the cross-sectional study of data from the annual health examinations of a medical center in Taiwan for the general population, a novel indicator, FMR, was suggested as an excellent body composition index for predicting the presence of MetS, prediabetes, DM and HTN. FMR was significantly associated with adverse health outcomes and a substantial dose dependent effect was noted in both genders. Furthermore, FMR-incorporated MetS had better predictive ability for the Framingham risk score than other definitions, particularly in females, indicating the possibility that FMR might have the potential capacity for predicting the incident risks of cardiovascular disease mortality.

In a Korean study composed of 264 adults, an increased visceral fat-to-thigh muscle ratio was significantly associated with MetS with an OR of 6.72 (95%CI = 1.60–28.14)[13]. Another finding obtained from a Korean cohort study indicated that the ratio of skeletal muscle mass to visceral fat was associated with MetS with an OR of 5.43 (95%CI = 2.56–13.34)[14]. Ezch et al. demonstrated that adverse body composition characterized by the ratio of whole body fat to lean mass was independently associated with metabolic dysfunction in women with polycystic ovary syndrome[15]. Compared to the above different body composition indices, our findings suggested that FMR was a useful indicator for predicting the presence of MetS, prediabetes, DM and HTN in the general population. To the best of our knowledge, the present study was the first to propose that FMR was strongly associated with adverse health outcomes in both males and females in a large-scale cross-sectional observational study.

Accumulated evidence has supported the relationship between fat mass and cardiometabolic outcomes. The distribution of body fat is associated with MetS in elderly adults, especially those with normal body weight[16]. In a longitudinal cohort study, those with more visceral fat had higher risks for developing incident MetS during a five-year follow-up[17]. Neeland et al. demonstrated that a higher amount of visceral fat was more useful in predicting the incident prediabetes and DM than other indices in a longitudinal study[5]. Visceral fat was considered an important predictor of insulin resistance in the non-diabetic population[18]. In a cohort study of 903 normotensive participants examining the development of HTN, visceral adipose tissue was associated with incident hypertension (relative risk: 1.22; 95%CI: 1.06–1.39) after multivariable adjustment[19]. Collectively, the above results were consistent with our findings that increased fat mass was associated with the presence of MetS, prediabetes, DM and HTN. Several studies have proposed the important role of fat tissue in cardiometabolic risks through different pathways. Dysfunction in adipose tissue, such as excessive free fatty acid metabolism changes, was caused by fat tissue accumulation[20]. Adipose alternation might lead to the impairment of hepatic metabolism[21]. It could also contribute to degradation of insulin, reduced degradation of apolipoprotein B, and increased hepatic glucose production, leading to hyperinsulinemia, hypertriglyceridemia and eventually DM[22, 23]. Another mechanism was the inflammation of adipose tissue caused by adipocyte hypertrophy, adipose tissue stresses and apoptosis[24]. Impaired insulin sensitivity and deteriorated glucose and lipid metabolism were related to adipocyte hypertrophy, which was described as a predominant and large volume of adipose tissue[25]. Increasing the secretion of chemoattractants and proinflammatory cytokines, such as MCP-1, TNF-α, IL-1, and IL-6, caused by adipocyte hypertrophy contributed to immune cell infiltration[26]. Increased numbers of macrophages caused by phenotypic switching were related to adaptive immune systems[27]. Changes in T-cell phenotype and the recruitment of B cells and T cells preceded macrophage infiltration[28]. A series of inflammatory changes in adipose tissue induced a chronic inflammation strongly implicated in the mechanisms underpinning whole-body metabolic dysregulation.

A progressive loss of muscle mass and an increment of fat mass were prevalent in the aging process. Excessive loss of appendicular lean mass was associated with Type 2 DM in community-dwelling older adults, particularly undiagnosed cases[7]. An inverse association was found between skeletal muscle mass with insulin resistance and the risk of prediabetes. In a recent Taiwanese study composed of 394 middle-aged and elderly adults, lower muscle mass was associated with the risk of metabolic syndrome, especially in the aging female population[6]. Emerging studies have proposed an association between sarcopenia and metabolic dysfunction. Chung et al. reported that the sarcopenic obese group showed close associations with insulin resistance, MetS, and cardiovascular disease risk factors in the elderly population[29]. Subjects with sarcopenia obesity were considered to have a greater risk of hypertension than simply obesity[30]. The significant associations between sarcopenia, defined in terms of muscle mass, sarcopenic obesity and MetS were observed in both men (RR = 1.31, 95%CI = 1.10–1.56) and women (RR = 1.17, 95%CI = 1.10–1.25)[3]. The mechanisms of the relationship between muscle mass and cardiometabolic risks were unclear. There were several plausible explanations, as follows. As an organ of an insulin-responsive target, the loss of muscle mass contributed to insulin resistance, MetS and HTN[31]. Levels of HOMA-IR were higher in sarcopenia participants than in control subjects[30]. The pathophysiology of DM caused an atrophy of muscles and included declines in the activity of anabolic hormones (*e*.*g*. IGF-I, testosterone, ghrelin)[32], and increased protein degradation caused by elevated expression of acrogens[33]. The reported loss of lean mass was caused by decreased responsiveness to insulin for the stimulation of muscle protein synthesis and for inhibiting protein breakdown[34]. Macrophage infiltration, one of the potential pathways of adipose dysfunction, was also related to inflammation in muscle mass[35]. Increased levels of IL-6 and CRP induced by elevated numbers of macrophages were significantly associated with the loss of total appendicular lean muscle mass[36]. Elderly adults with higher inflammatory levels such as TNF-α revealed the strongest associations and might be important markers of loss of muscle mass and strength[37]. In a recent study, the negative effects of CRP on muscle mass were identified by a reduction in the size of human myotubes along with a reduction in muscle protein synthesis[38]. Increased CRP levels reduced the phosphorylation of Akt, the major upstream regulator of the mTOR cascade involved in the regulation of muscle growth, and contributed to the impairment of muscle protein synthesis[39]. Another pathway was CRP-mediated cellular energy stress that increased the upregulation of AMPK, leading to the suppression of mTORC1 activity[40].

Interestingly, the gender difference is noted in the association between different definitions of MetS and Framingham risk score in the present study. FMRMetS is more closely associated with the risk score than MestS in females, but not in males. Several studies have reported that females have substantially greater body fat percentage, while males have greater visceral fat[41, 42]. This difference might be associated with the sexual dimorphism of body fat distribution and sex hormones[43].

The strengths of our study were a large population-based survey, and we proposed novel findings for the effect of a body composition index on cardiometabolic events. However, there were several potential limitations among our study. First, causal inference was not suitable because the present study was a cross-sectional design; thus, we could not explain whether FMR affected metabolic dysfunction. Second, the data for insulin resistance and HOMA-IR were not accessible in the health examination. If we could examine the association between insulin resistance and fat and muscle, interesting findings could be uncovered. Third, BIA is quite variable and it is not regarded by many as providing an accurate measure of body composition. Dehydration is an important factor affecting accuracy of BIA measurement that it causes an increase in the body's electrical resistance and an overestimation of body fat[44]. Exercise before BIA measurement contributes to an underestimation of body fat percentage and overestimation of fat-free mass because of reduced impedance[45]. Next, the information regarding drug use for DM, HTN, and dyslipidemia is not available in the study because these data is not assessing in the health examinations that may confound findings. Finally, the dataset was derived from only an Asian population. Thus, the limited ethnicity diversity in the participants might not reflect the association between FMR and metabolic risk factors in terms of racial differences.

## Conclusion

The present study highlighted a significant association between FMR and MetS, prediabetes, DM and HTN. FMR might be incorporated in newly constructed MetS definitions, which were better able to predict the incident cardiovascular risks than traditional criteria. We provided a simple and useful body composition indicator for the early prevention and management of cardiometabolic risks and improvement of public health. Further studies should focus more effort on the underlying mechanisms of the interaction between body composition and metabolic alternation.

## Supporting information

S1 Table Association between fat-muscle ratio with individual MetS components. (DOCX)

S2 Table Association between the CRP and different definitions of MetS. (DOCX)
