# Supplementary material for: Fat-to-muscle ratio is a useful index for cardiometabolic risks: A population-based observational study
Source: PLoS One. 2019 Apr 9;14(4):e0214994. doi: 10.1371/journal.pone.0214994 (PMC6456204; doi:10.1371/journal.pone.0214994)
Supplement: S1 Table — (DOCX) [file pone.0214994.s001.docx]

**Supporting Information Table 1. Association between fat-muscle ratio with individual MetS components**

| **Sex** | **Variable** | **Model ^a^ 1**  **β^b^ (95% CI)** | ***P***  **Value** | **Model** ^a^ **2**  **β^b^ (95% CI)** | ***P***  **Value** | **Model** ^a^ **3**  **β^b^ (95% CI)** | ***P***  **Value** |
| --- | --- | --- | --- | --- | --- | --- | --- |
| **Male** | **SBP** | 10.06 (7.57-12.54) | <0.001 | 8.44 (5.93-10.95) | <0.001 | 8.37 (5.86-10.88) | <0.001 |
|  | **DBP** | 7.20 (5.48-8.93) | <0.001 | 5.72 (3.98-7.46) | <0.001 | 5.84 (4.10-7.58) | <0.001 |
|  | **WC** | 14.08 (12.77-15.38) | <0.001 | 13.35 (12.05-14.65) | <0.001 | 13.50 (12.20-14.80) | <0.001 |
|  | **TG** | 53.38 (38.49-68.27) | <0.001 | 31.79 (17.34-46.24) | <0.001 | 34.44 (20.07-48.81) | <0.001 |
|  | **HDL** | -8.06 (-9.76, -6.37) | <0.001 | -8.28 (-9.97, -6.58) | <0.001 | -8.26 (-9.95, -6.58) | <0.001 |
|  | **FPG** | 11. 19 (7.12-15.26) | <0.001 | 10.90 (6.77-15.03) | <0.001 | 11.00 (6.87-15.13) | <0.001 |
| **Female** | **SBP** | 5.51 (3.62-7.39) | <0.001 | 4.25 (2.34-6.16) | <0.001 | 4.19 (2.29-6.10) | <0.001 |
|  | **DBP** | 3.15 (1.91-4.39) | <0.001 | 2.33 (1.07-3.59) | <0.001 | 2.34 (1.08-3.59) | <0.001 |
|  | **WC** | 6.72 (5.75-7.68) | <0.001 | 5.57 (4.63-6.50) | <0.001 | 5.60 (4.66-6.54) | <0.001 |
|  | **TG** | 18.65 (11.91-25.40) | <0.001 | 10.84 (4.16-17.52) | <0.001 | 11.07 (4.38-17.75) | <0.001 |
|  | **HDL** | -5.49 (-7.08, -3.90) | <0.001 | -4.24 (-5.73, -2.74) | <0.001 | -4.23 (-5.73, -2.74) | <0.001 |
|  | **FPG** | 6.02 (3.71-8.34) | <0.001 | 4.42 (2.08-6.76) | <0.001 | 4.52 (2.17-6.86) | <0.001 |

^a^ Adjusted covariates:

Model 1 = age

Model 2 = Model 1 + proteinuria, TC, UA, Cr, AST, albumin, hsCRP

Model 3 = Model 2 + history of smoking, drinking

**Supporting Information Table 2. Association between the CRP and different definitions of MetS**

|  |  | **Model** ^a^ **1**  **β^b^ (95% CI)** | ***P***  **Value** | **Model** ^a^ **2**  **β^b^ (95% CI)** | ***P***  **Value** | **Model** ^a^ **3**  **β^b^ (95% CI)** | ***P***  **Value** |
| --- | --- | --- | --- | --- | --- | --- | --- |
| Male | **MetS** | 0.054 (0.018-0.090) | 0.003 | 0.042 (0.006-0.078) | 0.022 | 0.036 (0.000-0.072) | 0.053 |
|  | **FMRMetS** | 0.064 (0.023-0.104) | 0.002 | 0.055 (0.015-0.096) | 0.007 | 0.050 (0.010-0.090) | 0.015 |
|  | **FMR + MetS** | 0.066 (0.022-0.110) | 0.003 | 0.050 (0.006-0.094) | 0.027 | 0.045 (0.000-0.089) | 0.048 |
| Female | **MetS** | 0.201 (0.162-0.240) | <0.001 | 0.171 (0.131-0.211) | <0.001 | 0.170 (0.131-0.210) | <0.001 |
|  | **FMRMetS** | 0.173 (0.129-0.217) | <0.001 | 0.151 (0.107-0.196) | <0.001 | 0.148 (0.104-0.193) | <0.001 |
|  | **FMR + MetS** | 0.199 (0.152-0.246) | <0.001 | 0.172 (0.125-0.220) | <0.001 | 0.170 (0.123-0.217) | <0.001 |

^a^ Adjusted covariates:

Model 1 = age

Model 2 = Model 1 + proteinuria, TC, UA, Cr, AST, albumin

Model 3 = Model 2 + history of smoking, drinking
