# Supplementary material for: Fat-to-muscle ratio is a useful index for cardiometabolic risks: A population-based observational study
Source: PLoS One. 2019 Apr 9;14(4):e0214994. doi: 10.1371/journal.pone.0214994 (PMC6456204; doi:10.1371/journal.pone.0214994)
Supplement: S2 Table — (DOCX) [file pone.0214994.s002.docx]

**Supporting Information Table 2. Association between the CRP and different definitions of MetS**

|  |  | **Model** ^a^ **1**  **β^b^ (95% CI)** | ***P***  **Value** | **Model** ^a^ **2**  **β^b^ (95% CI)** | ***P***  **Value** | **Model** ^a^ **3**  **β^b^ (95% CI)** | ***P***  **Value** |
| --- | --- | --- | --- | --- | --- | --- | --- |
| Male | **MetS** | 0.054 (0.018-0.090) | 0.003 | 0.042 (0.006-0.078) | 0.022 | 0.036 (0.000-0.072) | 0.053 |
|  | **FMRMetS** | 0.064 (0.023-0.104) | 0.002 | 0.055 (0.015-0.096) | 0.007 | 0.050 (0.010-0.090) | 0.015 |
|  | **FMR + MetS** | 0.066 (0.022-0.110) | 0.003 | 0.050 (0.006-0.094) | 0.027 | 0.045 (0.000-0.089) | 0.048 |
| Female | **MetS** | 0.201 (0.162-0.240) | <0.001 | 0.171 (0.131-0.211) | <0.001 | 0.170 (0.131-0.210) | <0.001 |
|  | **FMRMetS** | 0.173 (0.129-0.217) | <0.001 | 0.151 (0.107-0.196) | <0.001 | 0.148 (0.104-0.193) | <0.001 |
|  | **FMR + MetS** | 0.199 (0.152-0.246) | <0.001 | 0.172 (0.125-0.220) | <0.001 | 0.170 (0.123-0.217) | <0.001 |

^a^ Adjusted covariates:

Model 1 = age

Model 2 = Model 1 + proteinuria, TC, UA, Cr, AST, albumin

Model 3 = Model 2 + history of smoking, drinking
